# Measurement of renal cortex perfusion: A direct comparison of arterial spin labelling magnetic resonance imaging and [ ^15^O]H_2_O positron emission tomography

**DOI:** 10.1002/mrm.30638

**Published:** 2025-07-17

**Authors:** Naja Enevold Olsen, Christian Østergaard Mariager, Maibritt Meldgaard Arildsen, Sebastian Nielsen, Mikkel Holm Vendelbo, Michael Pedersen, Christoffer Laustsen, Steffen Ringgaard, Lars Poulsen Tolbod, Niels Henrik Buus

**Affiliations:** ^1^ Department of Nuclear Medicine & PET Aarhus University Hospital Aarhus Denmark; ^2^ Department of Nuclear Medicine Aalborg University Hospital Denmark; ^3^ Comparative Medicine Lab Aarhus University Aarhus Denmark; ^4^ Department of Renal Medicine Aarhus University Hospital Aarhus Denmark; ^5^ Department of Biomedicine Aarhus University Aarhus Denmark; ^6^ MR Research Centre Aarhus University Aarhus Denmark

**Keywords:** arterial spin labeling, positron emission tomography, renal perfusion magnetic resonance imaging, reproducibility

## Abstract

**Purpose:**

Reliable information about renal blood supply is important to understand kidney physiology and diseases. Arterial spin labeling MR (ASL‐MR) imaging and [^15^O]H_2_O positron emission tomography (PET) can noninvasively measure tissue perfusion but have never been directly compared. Using a hybrid PET/MR scanner, we performed simultaneous cortex perfusion measurements to assess repeatability and reproducibility of both modalities and establish their mutual correlation.

**Methods:**

Ten healthy subjects (mean 25 years, 5 males) with normal glomerular filtration rate were examined twice, 16 days (range 12–23) apart. Repeatability was assessed on Day 1 with two successive examinations. A single scan on Day 2 was used to assess reproducibility.

**Results:**

Single‐kidney perfusion varied between individuals from 150 to 422 mL/min/100 mL for ASL‐MR and from 184 to 470 mL/min/100 mL for PET. Repeatability and reproducibility were comparable between ASL‐MR and PET. Bias was generally low (−13 to 5 mL/min/100 mL), but 95% limits of agreement (LoAs) were wide, ranging from 69 to 88 mL/min/100 mL. Overall, correlations between ASL‐MR and PET perfusion values were weak, and in a linear mixed‐effects model, bias was 18 and LoA 136 mL/min/100 mL. Agreement between ASL‐MR and PET was acceptable at perfusion values between approximately 250 and 350 mL/min/100 mL. At lower perfusion, PET exceeded ASL‐MR, whereas the opposite was observed at higher perfusion.

**Conclusion:**

ASL‐MR and [^15^O]H_2_O PET renal cortical perfusion show comparable repeatability and reproducibility. Although perfusion obtained with the two modalities overall correlate weakly, there is an acceptable agreement in the mid‐physiological range.

## 1 INTRODUCTION

Sufficient kidney perfusion is essential for maintaining normal renal functions, glomerular filtration, and avoiding hypoxia.^1^, ^2^ Several methodologies exist to quantify total renal blood flow or parenchymal perfusion (blood flow per tissue volume), but they are seldom used in clinical practice, being either technically too complicated, involving contrast agents, or vascular catheterization.^3^ During the recent decades, assessment of renal tissue perfusion at the single‐kidney level has become available with MRI without the use of MR contrast media. MR scanners are widely available, and renal MR is increasingly used as a research tool to study alterations in renal hemodynamics associated with acute or chronic kidney diseases (CKDs).^4^ Despite optimism that information about MR‐derived renal perfusion or other parameters can be used to predict outcomes in patients with CKD^5^, ^6^ or a transplanted kidney,^7^ renal MR remains to become part of the diagnostic work‐up.

Arterial spin labeling (ASL) is the most promising MRI technique for absolute quantification of renal tissue perfusion.^8^ By using the arterial blood as an endogenous tracer,^9^ ASL can be performed without exogenous tracers or contrast agents. Furthermore, the method can be used at severely reduced kidney function, and due to relatively short scan times, it seems well‐suited for serial monitoring of perfusion for instance to test novel treatment regimens in CKD patients.

One of the challenges with ASL‐MR‐based perfusion measurements is the lack of validation and a paucity of direct comparisons to other techniques and especially methodologies based on exogenous tracers and proper kinetic models with determination of single‐kidney perfusion. Positron emission tomography (PET) is used extensively to quantify perfusion of the brain and myocardium but can also be used to measure renal perfusion, with the first studies published already in 1993.^10^, ^11^ PET investigations of renal perfusion in humans remain relatively few.^12^ Most studies used [^15^O]H_2_O, which is a freely diffusible and chemically inert tracer that is rapidly extracted by the tissue and with very limited urinary excretion, rendering it possible to use a simple one‐compartment tissue model.^13^ However, the accuracy may be influenced by the quality of image‐based input function measurements, limited spatial resolution, partial volume, and spill‐over effects.

Renal perfusion is regulated by hormonal, hemodynamic, and autonomic nervous system factors as well as intrarenal feedback mechanisms that can change rapidly from one situation to another.^14^, ^15^, ^16^ An optimal comparison of methods determining renal perfusion should therefore be based on simultaneous measurements. As perfusion is larger in the cortical than in the medullary parts of the kidney, a useful comparison should also be based on the same tissue, which again should be representative of the entire kidney.

In the present study, we used a hybrid PET/MR scanner to perform simultaneous ASL‐MR and [^15^O]H_2_O PET measurements of renal cortical perfusion to establish short‐term (intravisit) repeatability and longer‐term reproducibility of both methods and to explore their mutual correlation.

## 2 METHODS

### 2.1 Participants

**FIGURE 1:**
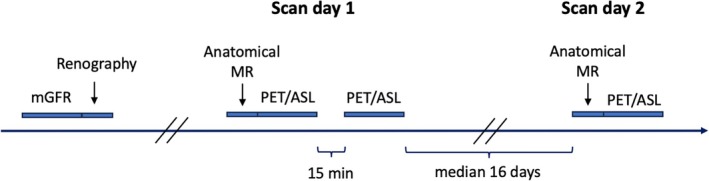
Illustration of the examinations in the study with measured glomerular filtration rate (mGFR) and renography followed later by two positron emission tomography (PET)/arterial spin labeling (ASL)–MR scan days separated by a median of 16 days. The Day 1 scan included two repeated PET/ASL scans performed 15 min apart, whereas the Day 2 scan included one PET/ASL scan. See text for further details.

Young healthy volunteers were recruited through an advertisement and underwent biochemical screening (including plasma choriogonadotropin for female participants) before inclusion. The study was performed in line with the principles of the Declaration of Helsinki and approved by the Committee on Biomedical Research Ethics for Central Denmark Region (Journal No. 1‐1072‐334‐20) with obtainment of written informed consent. All participants had a measurement of single kidney glomerular filtration rate (GFR) followed by PET/MR imaging, as explained subsequently and illustrated in Figure 1.

### 2.2 Single‐kidney GFR

GFR was assessed as plasma clearance of technetium‐99m DTPA (diethylenetriaminepentaacetic acid) with plasma samples 180, 200, 220 and 240 min after injection of 30 MBq of the tracer, measured on a gamma counter (2480 Wizard^2^; PerkinElmer, CT, USA). The GFR determination was immediately followed by injection of 100 MBq technetium‐99m MAG3 (mercaptoacetyltriglycine) for a renography (NephroCam; DDD‐Diagnostic, Denmark). Single‐kidney GFR was then determined as total GFR × relative perfusion distribution (%) based on the renography.

### 2.3 Renal imaging

We used a dual modality imaging protocol, executed on a 3T Signa PET/MR scanner (GE Healthcare, Milwaukee, WI, USA) equipped with a 16‐channel anterior and a 14‐channel posterior imaging array. PET/MR imaging was performed on two different days with a median interval of 16 days (range 12–23 days). As PET/MR scans were not always performed in the morning, the participants were not fasting. They avoided strenuous exercise in the hours before the scan and were asked to have a normal fluid intake but were not administered extra fluid to refrain them from feeling uncomfortable. The scan protocol is illustrated in Figure 1. On Day 1, baseline anatomical MR and functional PET/MR imaging were performed followed by a 15‐min break with the person taken out of the scanner. During the break, the person remained in the supine position on the scanner bed. Afterward, the functional PET/MR imaging protocol was repeated. On Day 2, the anatomical MR was repeated and followed by functional PET/MR imaging. The imaging protocols are further detailed subsequently.

Blood pressure (BP) and heart rate were measured just before commencing and immediately after termination of each PET/MR scan using a MR‐compatible automatic oscillometric device (Philips Expression MR400). The mean of the two measurements was used to monitor BP and heart rate during the scan.

### 2.4 Anatomical MRI

Anatomical MRI to assess the renal anatomy consisted of axial and coronal‐oblique T_1_‐weighted three‐dimensional liver acquisition with volume acceleration (LAVA) Flex, as well as coronal‐oblique T_2_‐weighted two‐dimensional fast spin echo. Imaging parameters for axial LAVA Flex were as follows: repetition time (TR) = 4.3 ms, echo times (TEs) per scan = 2, TE_1_ = 1.1 ms, TE_2_ = 2.2 ms, flip angle (FA) = 12°, field of view (FOV) = 480 × 384 mm^2^, acquisition matrix = 320 × 256, slice thickness = 3 mm, acquired with breath‐hold in one slab with 80 locations per slab. Imaging parameters for coronal‐oblique LAVA Flex were the same as for the axial acquisition, except for FOV = 480 × 480 mm^2^. The LAVA Flex acquisitions were used to reconstruct in‐phase and out‐of‐phase, as well as water and fat images. Imaging parameters for fast spin echo were TR = 5 s, TE = 102 ms, echo train length = 16, echo spacing (∆TE) = 7.9 ms, refocus FA = 111°, FOV = 380 × 380 mm^2^, acquisition matrix = 448 × 256, slice thickness = 4 mm, slice spacing = 0.4 mm, number of excitations = 5, fat saturation, acquired with respiratory triggering in 22 slices.

### 2.5 Functional PET/MR imaging

Functional imaging of the kidneys consisted of simultaneous coronal‐oblique two‐dimensional arterial spin labeling MR (ASL‐MR) and dynamic [^15^O]H_2_O PET acquisitions. ASL‐MR imaging was performed using multislice flow‐sensitive alternating inversion recovery (FAIR) ASL with background suppression. Parameters for FAIR ASL were TR = 7 s, TE = 19 ms, inflow time = 1500 ms, FOV = 380 × 380 mm^2^, acquisition matrix = 96 × 128, slice thickness = 5 mm, slice gap = 5 mm, 5 slices, 20 control/label pairs, acquired with free‐breathing using a single‐shot spin‐echo echo‐planar imaging readout.^8^ A proton density image was also acquired for each slice. Total scan time for the ASL acquisition was 4 min 54 s.

Simultaneously with the ASL acquisition, a dynamic [^15^O]H_2_O PET was acquired. A dose of 400 MBq [^15^O]H_2_O was diluted in 20 mL saline and injected in a cubital vein at a precise flow rate of 1 mL/s using a Medrad MRXperion MR injection system (Bayer AG, Leverkusen, Germany). The PET acquisitions each had a duration of 7 min and were reconstructed as a dynamic image series with 26 frames (1 × 10, 8 × 5, 4 × 10, 2 × 15, 3 × 20, and 8 × 30 s) in 2.8 × 2.8 × 2.8 mm^3^ isotropic voxels using the VuePointFX SharpIR algorithm with all common corrections and a 3‐mm Gaussian postfilter applied.

### 2.6 PET/MR data processing and analysis

Renal volume was estimated using a nnU‐Net deep learning model^17^ trained on publicly available annotated T_1_‐weighted MR images of the kidneys.^18^ The model was used to segment both kidneys of each participant, using the axial T_1_‐weighted in‐phase LAVA Flex data sets. The resulting renal contours were inspected and manually corrected, if necessary, followed by calculation of the right and left whole‐kidney volumes.

ASL‐MR images underwent motion compensation processing by rigid‐body affine transformation, separately for each repetition and kidney, before averaging and subtraction of label and control images. Representative examples of the resulting averaged perfusion‐weighted images can be found in Figure S1. Calculation of quantitative perfusion images was then performed using the algorithm implemented by the scanner vendor^19^ as explained in Supporting Information S1. The following parameters were used for the perfusion calculation: T_1,blood_ = 1600 ms, T_1,tissue_ = 1400 ms, partition coefficient = 0.9, combined efficiency = 0.6375. Both ASL motion compensation and perfusion image calculation were performed directly on the scanner reconstruction computer using vendor‐supplied algorithms (GE Healthcare).

**FIGURE 2:**
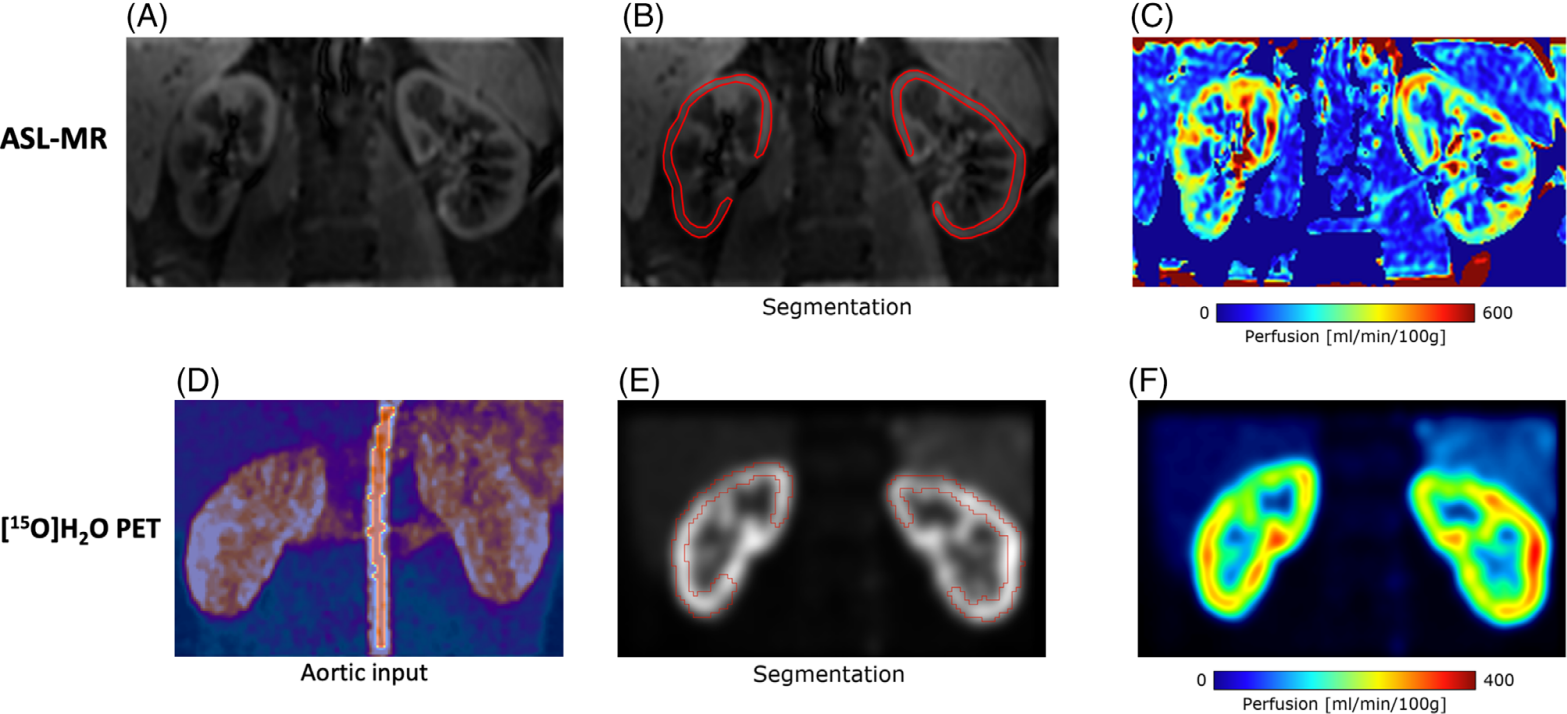
The top panel (A–C) shows a representative example of a renal arterial spin labeling (ASL)–MR scan with placement of regions of interest (ROIs) covering the cortex with the corresponding perfusion map. The bottom panel (D–F) shows the corresponding [^15^O]H_2_O positron emission tomography (PET) scan from the same person and scan sequence, with placement of ROIs covering the cortex with the corresponding perfusion map. (D) The image‐derived input function (IDIF) (the maximum intensity projection of summed dynamic PET with overlay of aorta mask used to extract IDIF; the mask was derived from cluster analysis). Note that the ASL‐MR pictures are acquired in a coronal‐oblique orientation, which depends on the slant of the kidneys, whereas the PET pictures are visualized in the coronal orientation. The pictures therefore show the same regions of the kidneys but at different angles (slice orientations).

Segmentation of the renal cortex was performed on the averaged perfusion‐weighted images and used to calculate global perfusion values, excluding voxels exceeding 500 mL/min/100 mL.^8^, ^20^ Data exclusion was performed based on visual assessment of the averaged perfusion‐weighted images. Whole slices were excluded from the segmentation in cases of motion compensation failure. Smaller image regions suffering from artifacts were also excluded from the segmentation. The segmentation was performed using in‐house‐developed software. A representative ASL‐MR example is depicted in Figure 2A, with regions of interest (ROIs) for ASL determination and the corresponding perfusion depicted in Figure 2B,C respectively. Representative examples of ASL data exclusion can be found in Figure S1. Among the total number of 60 ASL‐MR single‐kidney scans, only one scan contained only one slice of sufficient quality for determination of perfusion, corresponding to 1.7%. The percentages of kidneys analyzed using two, three, four, and five slices of sufficient quality for determination of perfusion were 8.3%, 16.7%, 25.0%, and 48.3%, respectively.

[^15^O]H_2_O PET scans were analyzed using a standard one‐tissue compartment model with two rate constants corresponding to uptake, K_1_, and clearance, k_2_, a variable time delay, and a fixed arterial blood volume (V_a_ = 0.15 mL/mL)^21^ using in‐house‐developed software based on *MATLAB* (version: 9.13.0 [R2022b]; The MathWorks Inc., Natick, MA, USA). Perfusion was estimated as the clearance rate, k_2_, multiplied by the physiological partition coefficient, *p* = 0.94 mL/g.^21^, ^22^ Only the first 150 s of the scan was used for analysis. The arterial blood input function for the model was derived from the abdominal aorta superior to the renal arteries using an automatic clustering method.^23^ The image‐derived arterial input function is illustrated in Figure 2D. The ROIs for determination of PET perfusion and the calculated perfusion image are illustrated in Figure 2E,F for the same person and the same scan as the ASL‐MR. Note that ASL data were acquired in a coronal‐oblique orientation, whereas PET data are visualized in the coronal orientation. This explains why contours of the kidneys differ between the ASL‐MR and PET pictures. Renal cortical ROIs were drawn on summed coronal images using PMOD Version 4.0 (PMOD Technologies LLC, Zürich, Switzerland). Parametric images of K_1_ were calculated using a basis function method.^23^

### 2.7 Data presentation and statistical analysis

Background data are presented as mean values with standard deviations (SDs) and range after testing for normal distribution using QQ plots. Comparison of BP and heart rate between two scans were performed using a paired t‐test. Individual single‐kidney cortex perfusion values are presented for all three scan sessions, and the perfusion of left‐sided and right‐sided kidneys are compared with an unpaired t‐test for all three scans. Simple linear regression with coefficients of determination (R^2^) are used to illustrate the association between perfusion values obtained at the same day (repeatability) and between days (reproducibility) for both ASL‐MR and PET. Equations for the linear associations are given as Y = a·X + b, with a representing the slope and b representing the intercept with the y‐axis. Repeatability and reproducibility data are furthermore illustrated by Bland–Altman plots with bias and 95% limits of agreement (LoAs).

The agreement between simultaneously obtained ASL‐MR and PET perfusion values are also illustrated at the single‐kidney level by simple linear regressions. These data are also presented using R^2^ values and equations in addition to Bland–Altman plots.

Due to repeated scans of the same individuals, the bias, LoA, and coefficient of variance (CoV) were also calculated using a linear mixed‐effects model (Stata 18.0; StataCorp LP, College State, TX, USA) with fixed effects of modality (ASL‐MR/PET) and scan sequence (one or two or three) and the interaction between them. Participant, kidney within participant, and day within kidney and participant were included as random effects. The SD corresponding to participant and day were allowed to vary with modality. LoAs were calculated as 1.96 times the square root of the sum of the relevant squared SDs. For example, when comparing measurements on different days for the same kidney with the same modality, the relevant SDs are those corresponding to between and within day. The SD used in the calculation of CoV was the same as the term used for determining the LoA divided by the square root of 2.

## 3 RESULTS

### 3.1 Participants

The clinical characteristics of the 10 participants are given in Table 1. They all had normal blood and urine biochemistry, and normal GFR as measured with technetium‐99 m DTPA. The distribution of GFR between the left and right kidneys was equal, demonstrating that all the scanned kidneys were healthy. Single kidney volumes are given as the mean of the two scan days. On average, the left kidneys were slightly larger than the right kidneys, but this was not significant. Total kidney volume was larger for males (370 ± 25 mL) as compared with females (263 ± 15 mL, *p* < 0.01).

**TABLE 1:** Clinical characteristics, biochemistry, GFR, and kidney volume of the participants.

	*N* or mean ± SD	Range
Males/females	5/5	
Age (years)	25.2 ± 3.2	21–31
Body mass index (kg/m^2^)	23.0 ± 2.1	19.7–24.5
Body surface area (m^2^)	1.9 ± 0.2	1.7–2.3
Active smokers/non‐smokers	2/8	
Biochemistry		
P‐creatinine (μmol/L)	78 ± 11	63–98
Estimated GFR (mL/min/1.73 m^2^)	106 ± 14	83–126
Urine albumin/creatinine (mg/g)	8 ± 7	1–22
B‐hemoglobin (mmol/L)	8.8 ± 0.9	7.5–10.7
P‐sodium (mmol/L)	141 ± 2	137–143
P‐potassium (mmol/L)	3.9 ± 0.3	3.6–4.6
P‐calcium ion (mmol/L)	1.26 ± 0.03	1.20–1.31
P‐phosphate (mmol/L)	1.14 ± 0.11	0.94–1.27
GFR (mL/min/1.73 m^2^)		
Measured GFR	106 ± 13	88–132
GFR, left kidney	53 ± 7	46–65
GFR, right kidney	53 ± 7	42–67
Kidney volume (mL)		
Left kidney	164 ± 34	122–211
Right kidney	152 ± 30	116–193

Abbreviations: GFR, glomerular filtration rate; SD, standard deviation.

BP increased significantly from Scan 1 (113 ± 11/59 ± 10 mmHg) to Scan 2 (118 ± 12/64 ± 9 mmHg) (*p* < 0.05), whereas heart rate remained unchanged (61 ± 9 vs 64 ± 9 beats per minute). At Scan 3, neither BP (110 ± 9/56 ± 10 mmHg) nor heart rate (56 ± 10 beats per minute) were different from the values at Scan 1.

### 3.2 Renal perfusion determined by ASL‐MR and [ ^15^O]H_2_O PET

**FIGURE 3:**
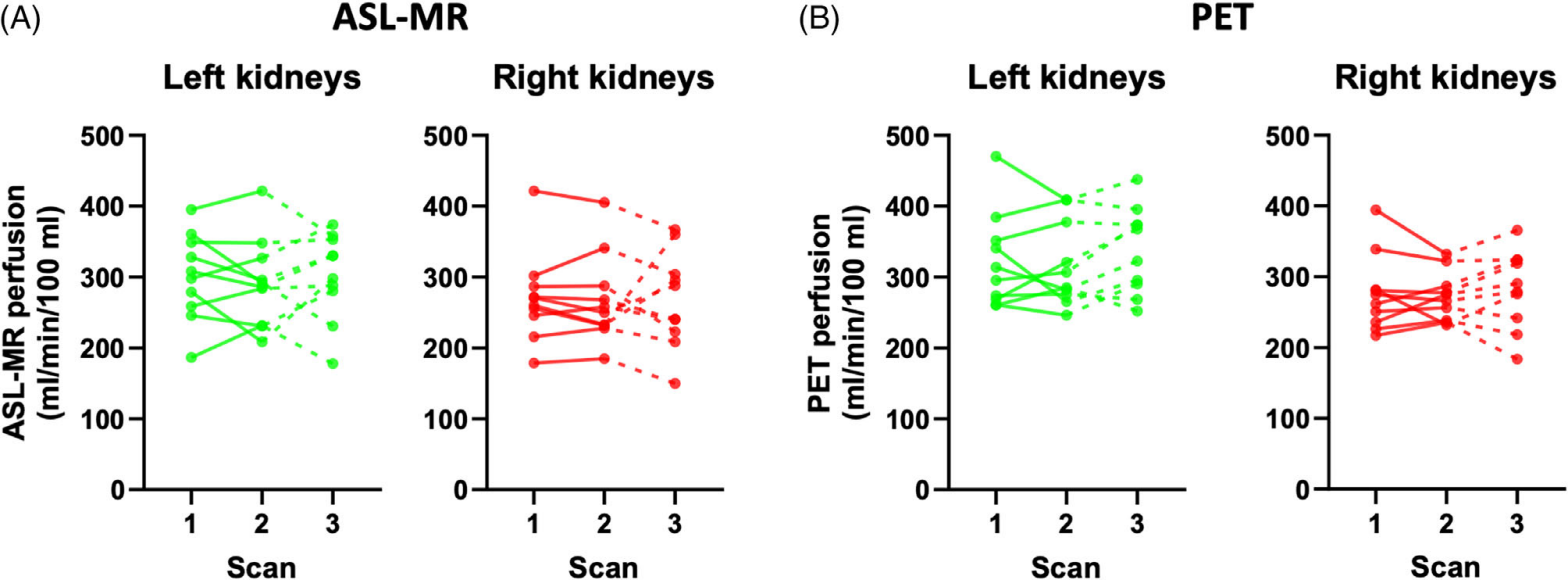
(A,B) Single‐kidney perfusion values from all three scan sessions. Data from arterial spin labeling (ASL) MR (A) and data from [^15^O]H_2_O positron emission tomography (PET) (B). Scans 1 and 2 represent the two PET‐MR scans performed on the first examination day, and Scan 3 the PET‐MR scan performed on the second examination day.

Individual single‐kidney values for ASL‐MR perfusion at the three scans are shown in Figure 3A, and the mean perfusion values are given in Table 2. There was a tendency for slightly higher perfusion values in the left as compared with the right kidneys during all scans, but it was not statistically significant. Individual single kidney values for [^15^O]H_2_O PET perfusion at the three scans are shown in Figure 3B, and the mean perfusion values are given in Table 2. The left kidney tended to have higher perfusion during all scans, although this was only significant for Scan 3.

**TABLE 2:** Single‐kidney cortical perfusion values determined by ASL‐MR and [^15^O]H_2_O PET.

			ASL‐MR	PET
			(mL/min/100 mL)	(mL/min/100 mL)
Day 1	Scan 1	Left kidney	301 ± 58	322 ± 65
		Right kidney	271 ± 60	276 ± 51
	Scan 2	Left kidney	293 ± 59	318 ± 57
		Right kidney	269 ± 60	272 ± 33
Day 2	Scan 3	Left kidney	302 ± 60	338 ± 58*
		Right kidney	268 ± 65	282 ± 52

*Note*: Data are presented as mean ± standard deviation. Abbreviations: ASL, arterial spin labeling; PET, positron emission tomography.

* *p* < 0.05 (left compared to right kidney).

**FIGURE 4:**
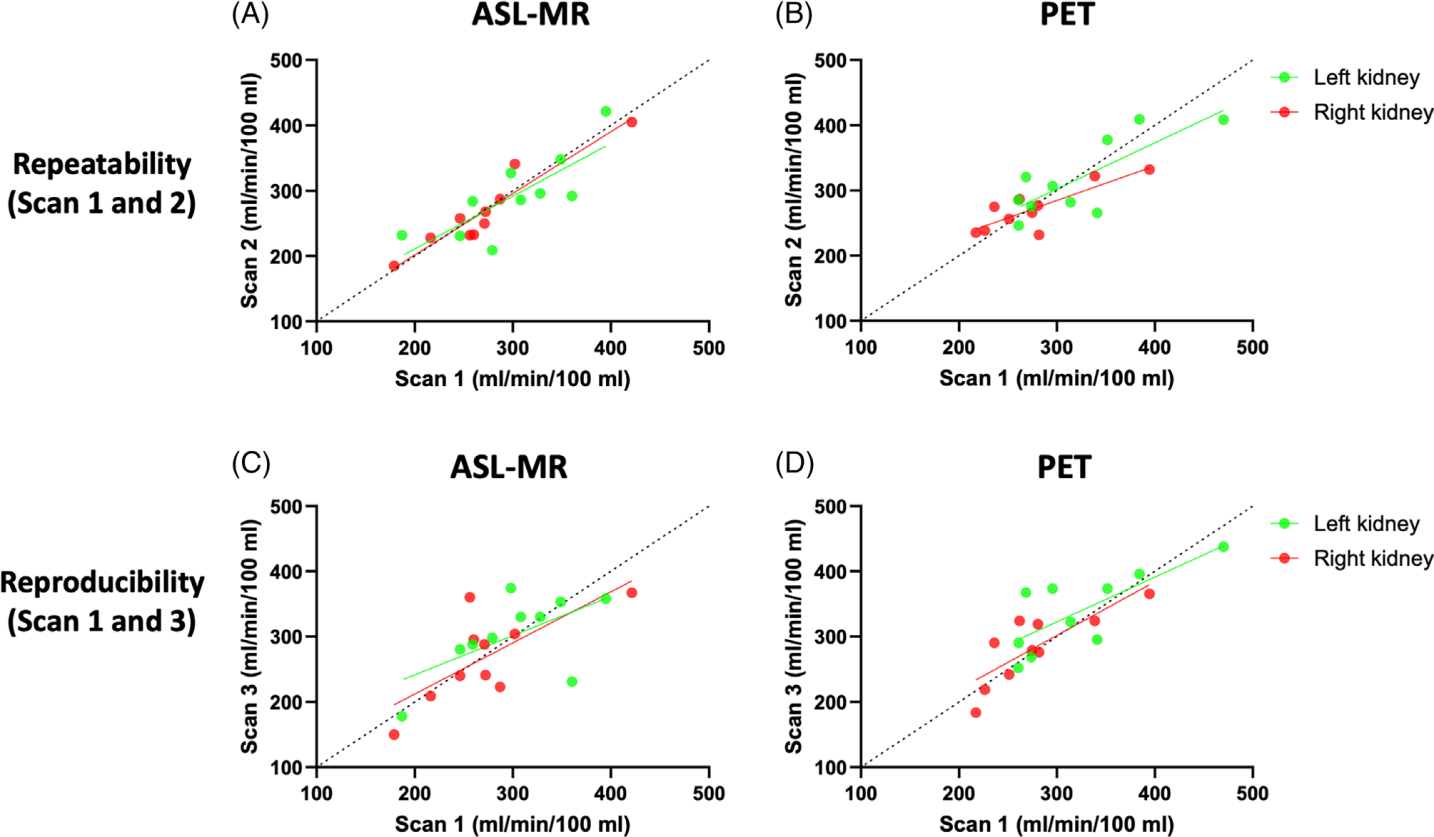
Repeatability (Scans 1 and 2) (A,B) and reproducibility (Scans 1 and 3) (C,D) of renal perfusion measured with arterial spin labeling (ASL) MR and [^15^O]H_2_O positron emission tomography (PET). The data are illustrated as linear associations separately for the left and right kidneys. Scans 1 and 2 represent the two PET‐MR scans performed on the first examination day and Scan 3 the PET‐MR scan performed on the second examination day. Equations and R^2^ values are as follows: Left kidneys: y = 0.81x + 50 (R^2^ = 0.62, *p* < 0.01) (A), right kidneys: Y = 0.94x + 14 (R^2^ = 0.90; *p* < 0.01). (B) Left kidneys: Y = 0.71x + 90 (R^2^ = 0.63; *p* < 0.01), right kidneys: Y = 0.53x + 126 (R^2^ = 0.69, *p* < 0.01). (C) Left kidneys: Y = 0.60x + 122 (R^2^ = 0.36; *p* = not significant), right kidneys: Y = 0.78x + 56 (R^2^ = 0.53, *p* < 0.05). (D) Left kidneys: Y = 0.69x + 117 (R^2^ = 0.57, *p* < 0.05), right kidneys: Y = 0.82x + 55 (R^2^ = 0.65; *p* < 0.01).

The repeatability data (Scans 1 and 2) at the single‐kidney level are illustrated in Figure 4A for ASL‐MR and in Figure 4B for [^15^O]H_2_O PET. The reproducibility data (Scans 1 and 3) at the single kidney level are depicted in Figure 4C for ASL‐MR and in Figure 4D for [^15^O]H_2_O PET. The equations revealed significant linear associations concerning both repeatability and reproducibility for both scan modalities. The equations for the linear associations had a slope below 1 and with a positive intercept with the y‐axis, but there were no major differences between the left and right kidneys regarding repeatability or reproducibility.

**FIGURE 5:**
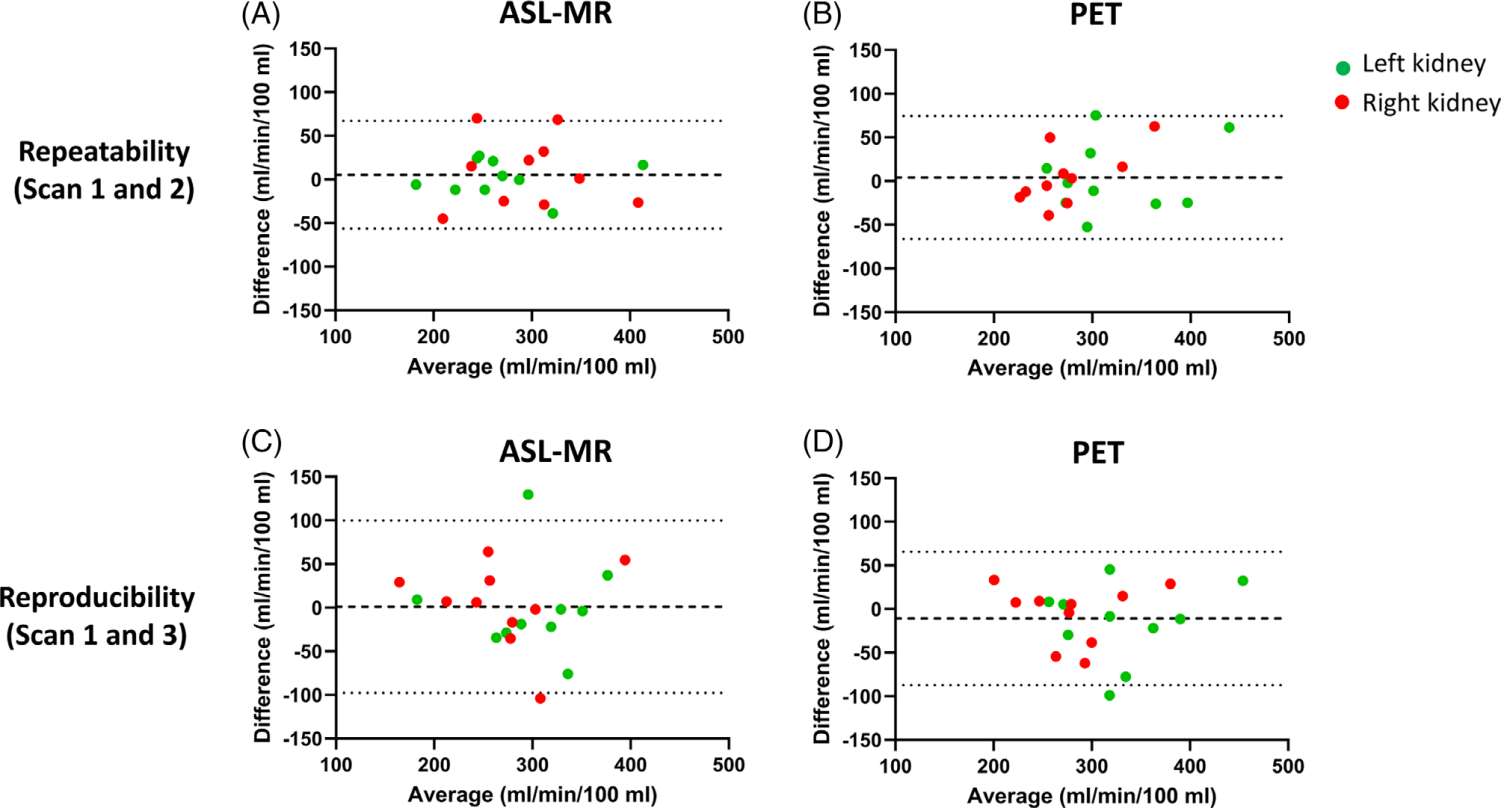
Bland–Altman plots of repeatability (Scans 1 and 2) (A,B) and reproducibility (Scans 1 and 3) (C,D) of renal perfusion measured with arterial spin labeling (ASL) MR and [^15^O]H_2_O positron emission tomography (PET), respectively. The dotted lines represent 95% limits of agreement (LoAs) for left and right kidneys combined. (A) Bias: 5, LoA: −56 – 67; (B) Bias: 4, LoA: −66 – 74; (C) Bias: 1, LoA: −98 – 100; (D) Bias: −11, LoA: −87 – 66 (all values in mL/min/100 mL).

The corresponding Bland–Altman plots are illustrated in Figure 5 with 95% LoA for the left and right kidneys combined. Figure 5A,B shows repeatability data for ASL‐MR and [^15^O]H_2_O PET, whereas Figure 5C,D shows reproducibility data for ASL‐MR and [^15^O]H_2_O PET, respectively. Bias was low (close to zero) for both repeatability and reproducibility, but LoAs were wide for and slightly larger for reproducibility than repeatability data. Of notice, the wide LoA for ASL‐MR reproducibility (Figure 5C) was driven by three data points. Two of these originate from the same individual who had considerably higher perfusion in both kidneys at Scan 3 as compared with Scan 1. Both scans were of good quality, and all five slices were used for the perfusion calculation. The third datapoint originates from another participant, in whom two and five slices were used in the perfusion calculation for Scans 1 and 3, respectively.

### 3.3 Bias, LoAs, and CoV based on linear mixed‐effects regression model

Bias, LoAs, and CoVs calculated from the linear mixed‐effects regression model are listed in Table 3 for ASL‐MR and PET perfusion. This analysis also revealed biases close to zero for both modalities and relatively wide LoA for both repeatability and reproducibility. However, both LoA and CoV seemed slightly lower for PET perfusion than ASL‐MR perfusion.

**TABLE 3:** Data from the linear mixed‐effects model concerning repeatability (Scans 1 and 2) and reproducibility (Scans 1 and 3) for each modality (ASL‐MR and [^15^O]H_2_O PET).

		Bias	LoA	CoV
		mL/min/100 mL	ml/min/100 mL	%
ASL‐MR	Repeatability	5.3	85.4	10.9
	Reproducibility	−1.6	88.5	11.2
PET	Repeatability	4.2	69.3	8.4
	Reproducibility	−12.9	73.2	8.7

Abbreviations: ASL, arterial spin labeling; CoV, coefficient of variance; LoA, 95% limits of agreement; PET, positron emission tomography.

### 3.4 Agreement between ASL‐MR and [ ^15^O]H_2_O PET renal perfusion

**FIGURE 6:**
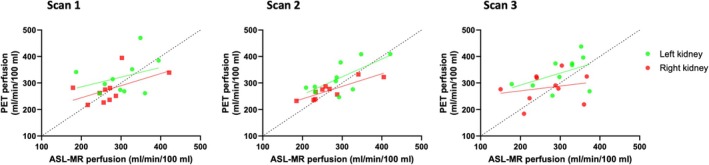
Linear associations between ASL‐MR and [^15^O]H_2_O PET perfusion values for Scan 1 (A), Scan 2 (B), and Scan 3 (C). The data are illustrated separately for the left and right kidneys. Equations and R^2^ values are as follows: Scan 1 left kidneys: Y = 0.37x + 212 (R^2^ = 0.11; *p* = not significant [NS]), right kidneys: Y = 0.46x + 153 (R^2^ = 0.29; *p* = NS). Scan 2 left kidneys: Y = 0.67x + 121 (R^2^ = 0.50; *p* < 0.05), right kidneys: Y = 0.47x + 145 (R^2^ = 0.75; *p* < 0.01). Scan 3 left kidneys: Y = 0.45x + 201 (R^2^ = 0.21, *p* = NS), right kidneys: Y = 0.18x + 234 (R^2^ = 0.05; *p* = NS).

Figure 6A–C shows the linear associations between the simultaneous ASL‐MR and PET perfusion measurements for each of the three PET‐MR scans illustrated separately for the left and right kidneys. The analysis revealed only very weak linear associations between perfusion values for the two modalities, which only reached significance during Scan 2. The equations of the linear associations showed that all slopes remained significantly below 1 with high y‐intercept values.

**FIGURE 7:**
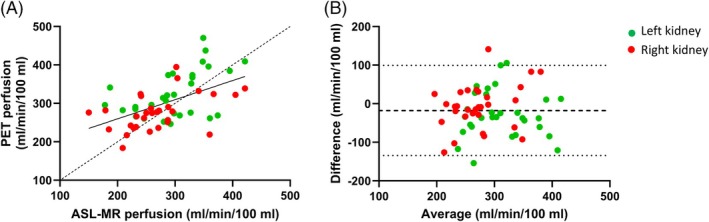
(A) Illustration of the linear association between arterial spin labeling (ASL) MR and [^15^O]H_2_O positron emission tomography (PET) perfusion based on data from all scans and all kidneys (*n* = 60, y = 0.50x + 160, R^2^ = 0.27; *p* < 0.01). (B) The corresponding Bland–Altman plot (bias: −18, limit of agreement: −134 to 99 mL/min/100 mL).

Figure 7A shows where all 60 single‐kidney perfusion measurements merged. This crude linear correlation has a slope of 0.50 (95% confidence interval 0.28–0.71) and R^2^ of 0.27. This means that a 100% variation in ASL‐MR perfusion is only mirrored by a 50% variation in [^15^O]H_2_O PET perfusion.

The agreement between ASL‐MR and PET perfusion was acceptable at values between approximately 250 and 350 mL/min/100 mL. At lower perfusion, PET values clearly exceeded ASL‐MR values, whereas the opposite was seen at higher perfusion values. The data are presented in a Bland–Altman plot in Figure 7B, demonstrating a bias of 18 mL/min/100 mL with wide 95% LoA.

Based on the linear mixed‐effects model, the bias was also found to be 18 mL/min/100 mL, whereas 95% LoA was 136 mL/min/100 mL and CoV was 17%.

### 3.5 Association between cortical perfusion and GFR

Figure S2 illustrates the association between cortex perfusion and single‐kidney GFR for ASL‐MR (Figure S2A) and [^15^O]H_2_O PET (Figure S2B), respectively. The perfusion value for each kidney represents the mean from Scans 1, 2, and 3 for each modality, and the linear associations are based on all 20 kidneys. The association between perfusion and GFR is weak and not significant for either ASL‐MR (R^2^ = 0.07, *p* = 0.25) or [^15^O]H_2_O PET (R^2^ = 0.16, *p* = 0.08).

## 4 DISCUSSION

The study presents a direct comparison of ASL‐MR and [^15^O]H_2_O PET‐based measurement of renal cortex perfusion using simultaneous measurements. The two methods demonstrated comparable repeatability and 2 weeks' reproducibility, but their mutual correlation was poor overall, and outside the mid‐physiological range, the two modalities showed very different perfusion values. Our data highlight the large temporal variations in renal perfusion as assessed by the same technique and that a large change must take place before it can be considered significant. Our data show this is the case for both ASL‐MR and PET.

### 4.1 ASL‐MR perfusion measurements

Many studies have used ASL‐MR in healthy subjects with normal GFR, reporting mean renal perfusion values from less than 200 to more than 400 mL/min/100 mL.^12^, ^24^ Several of these have also evaluated intravisit and intervisit reproducibility in terms of CoV being in the range of 3% to 18%, as recently reviewed by Odudu et al.^24^ The present data are in line with these findings both concerning cortex perfusion levels and reproducibility. Our MR protocol on Day 1 can to some extent be compared with a recent study in 12 individuals who were also scanned twice within a short time frame, showing excellent agreement between two successive scans.^25^ Removal from and reinsertion of the participant into the scanner, as in our experiment, may induce some distress and circulatory changes in the participant, which affects renal perfusion.

Longer‐term reproducibility is of more clinical relevance if the scans are used to evaluate the consequences of progressing kidney diseases or the effects of various treatments on renal perfusion.^26^ In this aspect, single‐kidney measurements are important, as some conditions, like renovascular disease, may affect the two kidneys differently. Even in healthy people, variation of renal perfusion can be considerably over time. As documented by our data and others, CoV is often large^24^ and LoA wide, approaching 50–100 mL/min/100 mL^27^, ^28^ when comparing two scans taken weeks or months apart.

The magnetic labeling of arterial blood water protons constitutes one of the main challenges with ASL. This infers a generally low signal‐to‐noise ratio, as the labeling effect of ASL on the image contrast is weak. Therefore, ASL acquisitions are performed with lower matrix sizes and with multiple signal repetitions, to yield an acceptable signal‐to‐noise ratio.^8^ This could be a problem in patients with reduced cooperation but is possibly less of an issue in the present study with selected young healthy participants with good cooperation.

In terms of postprocessing, the natural movement of the kidneys during the scan necessitates image registration before averaging individual repetitions. This is crucial to minimize residual motion artifacts before the subtraction of label and control images. Although automated segmentation for determination is ASL perfusion has been developed,^29^ this process is most often completely manual, and therefore also observer dependent.

### 4.2 PET perfusion measurements

Recently, Paivarinta et al. reviewed studies on renal perfusion using PET.^12^ Nearly all studies used [^15^O]H_2_O, and they report mean cortical perfusion values in healthy persons to be in the range of 2.7 to 4.7 mL/min/g. Our data thus fall within the lower end of this range.

Repeatability and reproducibility data of [^15^O]H_2_O PET perfusion are very sparse. Nitzsche et al. performed repeated [^15^O]H_2_O scans in 5 subjects 15 min apart, claiming an interstudy bias of only 2.2 ± 1.3%.^11^ In a later study, Alpert et al. scanned 5 persons with normal kidney function 7 days apart, and based on the individual perfusion values, the bias can be calculated to 16 mL/min/100 mL with LoA of 185 mL/min/100 mL,^30^ which is twice that found in the current study.

Respiratory motion can lead to a smearing of the PET signal and a reduction of the amplitude of the time activity curve. However, the reduction is constant, which means the shape of the time activity curve is hardly affected. Consequently, the clearance, k2, which is used in this study as a measure of perfusion, is quite insensitive to respiratory motion. The spleen is in close anatomical proximity to the left kidney and despite thorough placement of ROIs in the renal tissue, spill‐over from high spleen activity cannot be completely avoided. This can contribute to the slightly higher [^15^O]H_2_O PET perfusion values of the left‐sided kidney compared to the right‐sided kidney. The boundaries between cortex and medulla cannot be clearly defined on the PET image, and the limited resolution with [^15^O]H_2_O PET and activity spill‐over from the cortex to the medulla makes it unreliable to differentiate between cortical and medullary perfusion. Although this has been attempted previously by peeling of voxels at the cortical parts, this approach is not validated.^31^ We therefore decided only to evaluate cortical perfusion from the ASL‐MR scans, which is also the current recommendation.^8^ Indeed, isolated medullary perfusion is hard to measure with both PET and ASL‐MR, and the reported values differ much between studies.

### 4.3 Comparison of ASL‐MR and PET perfusion measurements

There is no gold‐reference standard for quantification of total or single‐kidney perfusion. Effective renal plasma flow can be estimated using clearance of para‐aminohippuric acid (PAH), and this method is used widely in research. PAH plasma clearance has previously been compared with ASL‐MR perfusion in patients with metabolic syndrome and normal GFR with only a modest correlation between the two measures.^32^ PAH clearance has also been compared with renal PET perfusion in healthy persons^30^ and patients with CKD.^33^ Although the two measures correlate to some extent, alterations in renal hemodynamics and blood flow induced by angiotensin‐converting enzyme inhibitor treatment are differently reflected by the two methods.^33^ Furthermore, a direct comparison in terms of perfusion per tissue volume unit is difficult and depends on methods for correction of total or cortical kidney volume. As opposed to PAH clearance, both PET and ASL‐MR must be considered independent of tubular function, rendering these techniques more suitable in CKD patients receiving medications interfering with tubular anion transporters.^30^ PAH clearance is therefore not suitable as a standard against which both ASL‐MR and PET can be compared.

ASL‐MR and PET have also been tested against the microsphere method for assessment of renal perfusion in anesthetized pigs. Although cortical perfusion with ASL‐MR tended to be lower than the blood flow determined with microspheres, the two methods demonstrated reasonable agreement up to 550 mL/min/100 mL.^34^ Likewise, a correlation has been established between renal perfusion determined by [^15^O]H_2_O PET and microspheres but with LoA reaching 90 mL/min/100 mL.^35^ Although the microsphere method has several drawbacks and is not suitable for use in humans, these studies show that both ASL‐MR and PET are able to reflect a broad range of renal perfusion.

Using a 3T PET/MR scanner, a recent study compared renal perfusion measured by ASL‐MR and the positron‐emitting tracer ^64^Cu‐ATSM in 5 healthy persons and 10 CKD patients who were analyzed together.^36^ The perfusion values obtained in that study were converted to whole kidney blood flow and therefore not directly comparable to ours, but also Nishikawa et al. noticed a clear tendency for larger perfusion values for ASL‐MR as compared with PET at high perfusion levels and the opposite at low perfusion values. Furthermore, their study demonstrated LoA between the two methods reaching 100 mL/min/100 mL, which is like our findings.

Simultaneous ASL‐MR and [^15^O]H_2_O PET perfusion of the brain has previously been performed in young healthy males.^37^ That study describes relatively poor correlations between ASL‐MR and PET in both the resting state and at states of lower and higher cerebral perfusion. However, when combining all perfusion values from all states in the same analysis, the slope of the correlation was very close to unity, suggesting that a true change in perfusion is equally captured by both methods.^37^ We did not perform interventions to decrease or increase renal perfusion and, hence, a similar analysis cannot be made from our data. Interestingly, while we found the slope of the correlation between renal ASL‐MR and PET perfusion to be less than 1, it was larger than 1 in the cerebral perfusion.

### 4.4 Limitations

Our study includes several limitations. Renal perfusion is influenced by nonmodifiable as well as modifiable factors,^16^ and the perfect study condition is difficult to achieve. Our subjects were not examined at the exact same time of the day, but all scans were performed between 10 AM and 3 PM to minimize circadian variations. For the same reason, the participants did not fast, and they were not on a fixed sodium diet. As caffeine has not been shown to affect renal blood flow, the participants were not restricted from drinking coffee.^16^ We deliberately avoided large extra fluid loads, as this could lead to unpleasant bladder distention with increasing BP and mental stress. Effects of medication is deemed very limited in our study, as the only drug intake was contraceptive pills in 2 females. Whether external factors could differentially influence ASL‐MR perfusion and PET perfusion is unknown, but we find it unlikely that the relationship between the two parameters (Figures 6 and 7) should be markedly changed by altering the way our study subjects were prepared for the PET/MR scans. However, increasing age may be associated with decreasing cortical perfusion, even in the absence of kidney diseases, and our findings cannot be directly extrapolated to older individuals or to patients with CKD.^16^, ^38^

The limited sample size is also a limitation, and more participants will reduce the influence of outliers on bias and LoA. Larger studies and preferably multicenter studies using different PET‐MR scanners are needed to substantiate our findings. Likewise, a longer interval between the scan days could have yielded different results with regard to reproducibility.

Due to the inherited differences in image acquisition between ASL‐MR (oblique longitudinal slices) and [^15^O]H_2_O PET (coronal slices), it is not feasible to identify ASL‐MR and PET cortex areas, which correspond exactly to each other. We could therefore not perform a direct comparison of perfusion values at a segmental level. Although image quality was overall satisfactory with nearly all kidneys contributing with 2 or more slices for the ASL‐MR determination of cortex perfusion, a limited number of voxels could still result in a datapoint becoming an outlier.

We primarily performed the single‐kidney GFR and volume measurements to ensure we studied healthy kidneys. The poor, between‐subjects, relation between single‐kidney GFR and cortical perfusion, whether determined by ASL‐MR or [^15^O]H_2_O PET, illustrates that factors beyond perfusion determine GFR.

### 4.5 Conclusions and implications

ASL‐MR and [^15^O]H_2_O PET renal cortical perfusion show comparable repeatability and reproducibility. Although perfusion obtained with the two modalities overall correlate weakly, there is an acceptable agreement in the mid‐physiological range. The relative overestimation with [^15^O]H_2_O PET at lower perfusion levels and a relative overestimation with ASL‐MR at high perfusion is a potential problem for implementation of these methods in clinical use. The reasons should be further addressed, including interventions extending renal perfusion to levels lower and higher than the normal physiological range. Such interventions could include systemic infusion of angiotensin II causing renal vasoconstriction^39^ or infusion of amino acids and dopamine causing renal vasorelaxation.^40^ Combined PET/MR studies are also necessary in CKD patients, and the ongoing iBEAt study in diabetic kidney disease includes both PET and ASL‐MR and will contribute important data to this.^41^

## Supporting information


Supporting Information S1.

**Figure S1.** Three representative examples of the image quality of the acquired arterial spin labeling MR (ASL‐MR) data. Images are perfusion weighted after motion compensation, subtraction of the label and control pairs, and after averaging. The slices included in the ASL analysis for a particular kidney are indicated with a green border, whereas omitted slices are indicated with a red border. Light blue arrows indicate smaller areas that were excluded from the region‐of‐interest (ROI) segmentation due to image artifacts.
**Figure S2.** Linear associations between cortex perfusion and single‐kidney glomerular filtration rate (GFR) for ASL‐MR (R^2^ = 0.07, *p* = 0.25) (A) and [^15^O]H_2_O PET (R^2^ = 0.16, *p* = 0.08) (B). The perfusion value for each kidney represents the mean from Scans 1, 2, and 3 for each modality and the linear associations are based on all 20 kidneys.

## Data Availability

The data sets generated and analyzed in the study are available from the corresponding author on reasonable request.
