# Supplementary material for: Measurement of renal cortex perfusion: A direct comparison of arterial spin labelling magnetic resonance imaging and [ 15O]H2O positron emission tomography
Source: Magn Reson Med. 2025 Jul 17;94(6):2537–49. doi: 10.1002/mrm.30638 (PMC12501728; doi:10.1002/mrm.30638)
Supplement: Supplementary file 1 — Supporting Information S1. [file MRM-94-2537-s001.docx]

**Supplementary information S1**

The equation used internally by the vendor software to calculate renal perfusion (RP) maps, as described previously by Alsop *et al* (1). Here, $\lambda$ is the blood-tissue partition coefficient, $\alpha$ is the inversion efficiency, $M^{0}$ is the equilibrium tissue magnetization, $M^{tag}$ is the tissue magnetization in the tagged experiment, $M^{ctrl}$ is the tissue magnetization in the control experiment, $T_{1ns}$ is the $T_{1}$ decay in the absence of off-resonance RF saturation, $\delta$ is the arterial transit time from the tagging plane to the imaged slice, $\omega$ is the delay between the end of tagging and image acquisition,$T_{1a}$ is the $T_{1}$ of arterial blood, $T_{1s}$ is the $T_{1}$ decay during application of a tagging pulse and $\delta_{a}$ is the time required for blood to flow from the tagging slice to the arteries within the vascular compartment.

$$RP= \frac{-\lambda\left( M^{tag}-M^{ctrl} \right)}{2\alpha M^{0}} C(T_{1ns},T_{1s},T_{1a},{\delta,\delta}_{a})$$

where

$$\frac{1}{C}= T_{1ns}\exp\left( {-\delta}/{T_{1a}} \right)[\exp\left( {\min\left( \delta-\omega,0 \right)}/{T_{1ns}} \right)$$

$$-\exp({-\omega}/{T_{1ns}})(1-{T_{1s}}/{T_{1ns})]}$$

$$+T_{1a}\left[ {\exp((\min\left( \delta_{a}-\omega,0 \right)-\delta_{a})}/{T_{1a}} \right)$$

$$-exp((\min\left( \delta-\omega,0 \right)-\delta)/T_{1a})]$$

1. Alsop DC, Detre JA. Reduced transit-time sensitivity in noninvasive magnetic resonance imaging of human cerebral blood flow. *J Cereb Blood Flow Metab.* 1996; 16: 1236-1249.
