# Supplementary material for: Measurement of renal cortex perfusion: A direct comparison of arterial spin labelling magnetic resonance imaging and [ 15O]H2O positron emission tomography
Source: Magn Reson Med. 2025 Jul 17;94(6):2537–49. doi: 10.1002/mrm.30638 (PMC12501728; doi:10.1002/mrm.30638)
Supplement: Supplementary file 2 — Figure S1. Three representative examples of the image quality of the acquired arterial spin labeling MR (ASL‐MR) data. Images are perfusion weighted after motion compensation, subtraction of the label and control pairs, and after averaging. The slices included in the ASL analysis for a particular kidney are indicated with a green border, whereas omitted slices are indicated with a red border. Light blue arrows indicate smaller areas that were excluded from the region‐of‐interest (ROI) segmentation due to image artifacts. Figure S2. Linear associations between cortex perfusion and single‐kidney glomerular filtration rate (GFR) for ASL‐MR (R2 = 0.07, p = 0.25) (A) and [15O]H2O PET (R2 = 0.16, p = 0.08) (B). The perfusion value for each kidney represents the mean from Scans 1, 2, and 3 for each modality and the linear associations are based on all 20 kidneys. [file MRM-94-2537-s002.pdf]

Figure S1

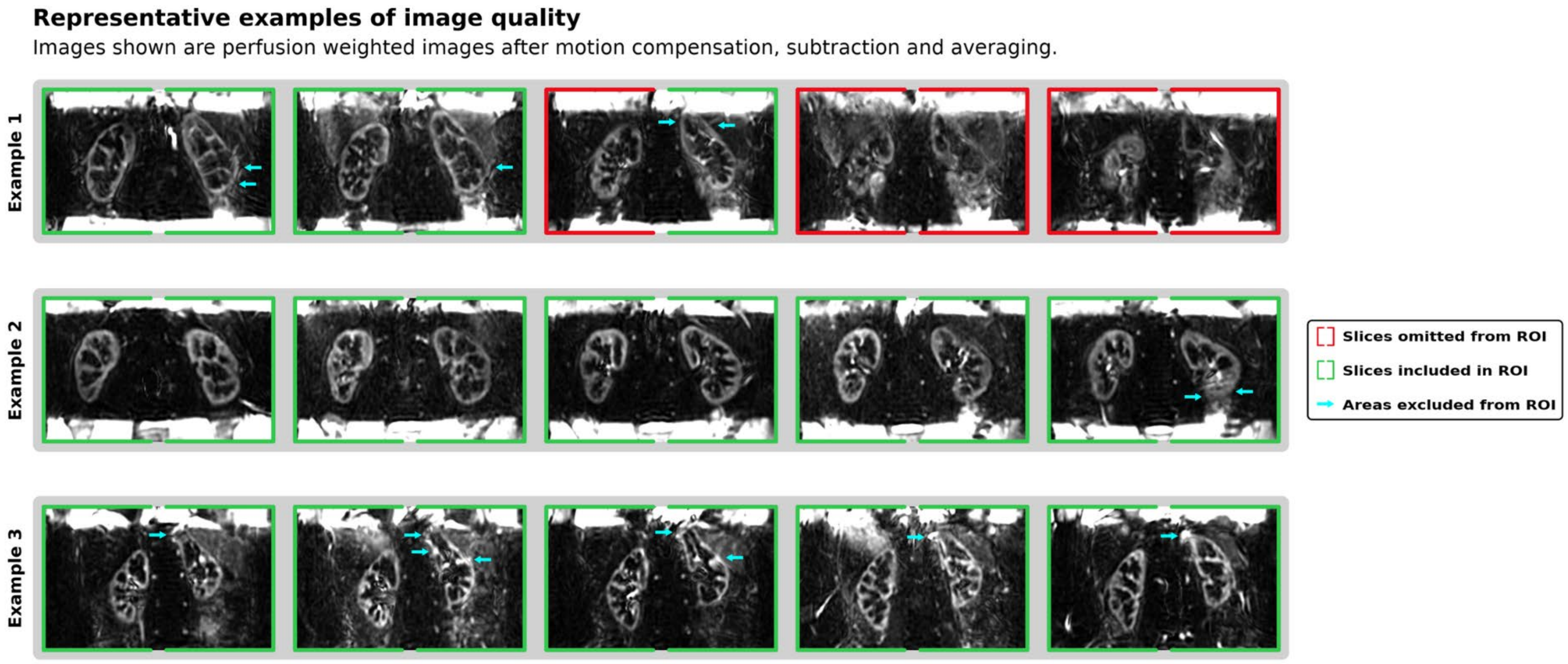

The figure shows 3 representative examples of the image quality of the acquired ASL-MR data. Images are perfusion weighted after motion compensation, subtraction of the label and control pairs, and after averaging. The slices included in the ASL analysis for a particular kidney are indicated with a green border, while omitted slices are indicated with a red border. Light blue arrows indicate smaller areas that were excluded from the ROI segmentation due to image artifacts.

Figure S2

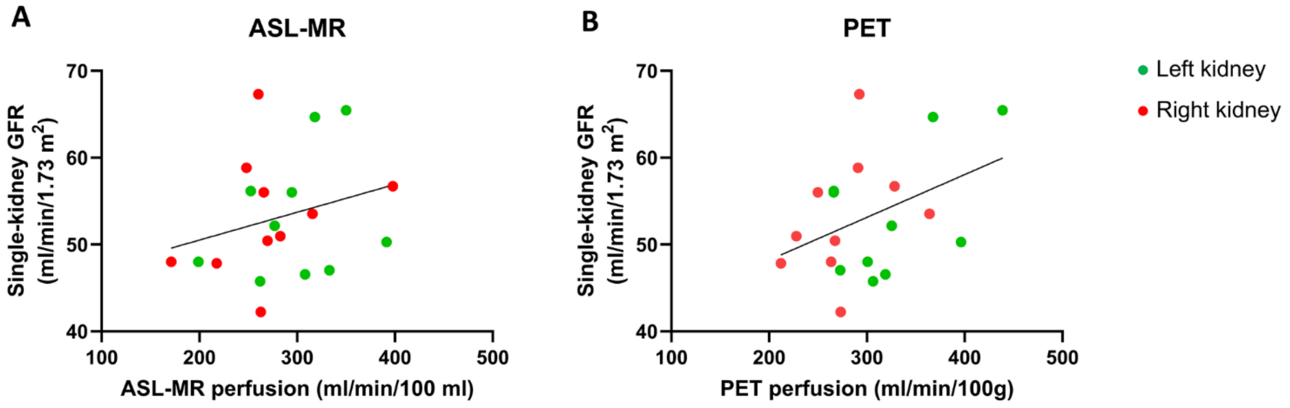

Linear associations between cortex perfusion and single-kidney GFR for **A**) ASL-MR (R^2=0.07, P=0.25) and **B**) [^{15}O]H_2O PET (R^2=0.16, P=0.08). The perfusion value for each kidney represents the mean from scans one, two and three for each modality and the linear associations are based on all 20 kidneys.
